# Carbohydrate sulfotransferase 3 (CHST3) overexpression promotes cartilage endplate‐derived stem cells (CESCs) to regulate molecular mechanisms related to repair of intervertebral disc degeneration by rat nucleus pulposus

**DOI:** 10.1111/jcmm.16440

**Published:** 2021-05-16

**Authors:** Yunzhi Guan, Chi Sun, Fei Zou, Hongli Wang, Feizhou Lu, Jian Song, Siyang Liu, Xinlei Xia, Jianyuan Jiang, Xiaosheng Ma

**Affiliations:** ^1^ Department of Orthopedics Huashan Hospital Fudan University Shanghai China

**Keywords:** carbohydrate sulfotransferase 3, cartilage endplate‐derived stem cells, intervertebral disc degeneration, migration, nucleus pulposus

## Abstract

To investigate the regulatory effect of carbohydrate sulfotransferase 3 (CHST3) in cartilage endplate‐derived stem cells (CESCs) on the molecular mechanism of intervertebral disc degeneration after nucleus pulposus repair in rats. We performed GO and KEGG analysis of GSE15227 database to select the differential genes CHST3 and CSPG4 in grade Ⅱ, Ⅲ and Ⅳ intervertebral disc degeneration, IHC and WB to detect the protein profile of CHST3 and CSPG4, Co‐IP for the interaction between CHST3 and CSPG4. Then, immunofluorescence was applied to measure the level of CD90 and CD105, and flow cytometry indicated the level of CD73, CD90 and CD105 in CESCs. Next, Alizarin red staining, Alcian blue staining and TEM were performed to evaluate the effects of CESCs into osteoblasts and chondroblasts, respectively, CCK8 for the cell proliferation of osteoblasts and chondroblasts after induction for different times; cell cycle of osteoblasts or chondroblasts was measured by flow cytometry after induction, and WB for the measurement of specific biomarkers of OC and RUNX in osteoblasts and aggrecan, collagen II in chondroblasts. Finally, colony formation was applied to measure the cell proliferation of CESCs transfected with ov‐CHST3 or sh‐CHST3 when cocultured with bone marrow cells, WB for the protein expression of CHST3, CSPG4 and ELAVL1 in CSECs, transwell assay for the migration of CESCs to bone marrow cells, TEM image for the cellular characteristics of bone marrow cells, and WB for the protein profile of VCAN, VASP, NCAN and OFD1 in bone marrow cells. CHST3 and CSPG4 were differentially expressed and interacted in grade Ⅱ, Ⅲ and Ⅳ intervertebral disc degeneration; CD73, CD90 and CD105 were lowly expressed in CESCs, osteogenic or chondroblastic induction changed the characteristics, proliferation, cell cycle and specific biomarkers of osteoblasts and chondroblasts after 14 or 21 days,; CHST3 affected the cell proliferation, protein profile, migration and cellular features of cocultured CESCs or bone marrow cells. CHST3 overexpression promoted CESCs to regulate bone marrow cells through interaction with CSPG4 to repair the grade Ⅱ, Ⅲ and Ⅳ intervertebral disc degeneration.

## 1 INTRODUCTION

Spinal degenerative disease is a common disease in spine surgery, and its incidence increases with the ageing, which has caused heavy family and society burden.^1^ The intervertebral disc is an avascular structure, and three special anatomical tissue components include the nucleus pulposus, the annulus fibrosus and the cartilage endplate.^2^ Intervertebral disc degeneration standards for the main reason of spinal degenerative diseases aetiology.^3^ The intervertebral disc composes of upper and lower cartilage endplates, jelly‐like nucleus pulposus and the outer fibrous ring, which is an annular layer of elastic fibres and fibrocartilage that surrounds the nucleus pulposus, and the internal cell nutrients are exchanged mainly through diffusion of nutrient channels on cartilage endplates.^4^ Cartilage endplate is a thin layer of hyaline cartilage of the adjacent vertebral body, and its calcification can cause diffuse dysfunction of the cartilage endplate, making the intervertebral disc unable to be orthopaedic regular substance exchange, imbalance in the homeostasis of the matrix metabolism in the disc, causing or accelerate disc degeneration.^5^ Many studies have confirmed cartilage endplate degeneration and intervertebral disc degeneration are closely related.^6^, ^7^ Therefore, the cartilage endplate degeneration mechanism becomes, in recent years, a new direction for the study of disc degeneration mechanism. How to repair calcified soft bone endplate, making it cartilage again, restore the normal material exchange of the disc replacement has also become an important topic in the biological treatment of disc degeneration.

Previous studies have confirmed the existence of endogenous stem cells in cartilage and endplate of intervertebral disc is a thin layer of cartilage, which also contains stem cells that regulate tissue homeostasis, namely cartilage endplate stem cells (CESCs).^8^, ^9^ In 2011, CESCs of intervertebral discs were first discovered by Liu et al.^10^ Compared with other exogenous stem cells, CESCs have better adaptability to the environment of the intervertebral disc and had a great potential to differentiate into chondrocytes.^11^ Therefore, CESCs were considered to have good application prospects. However, CESCs change their cellular characteristics during disc degeneration, which maybe one of the important mechanisms of disc degeneration; therefore, the study of CESCs in the intervertebral fate transition in the process of regression is of great significance. The cartilage endplate tissue has been proved to be an idea source to isolate cartilage endplate stem cells. The separated stem cells have different multipotential differentiation into adipocytes, osteoblasts and chondroblasts.^10^

Bioinformatic analysis of GSE15227 database shows the differential gene expression of CHST3 and CSPG4 in grade Ⅱ, Ⅲ and Ⅳ intervertebral disc degeneration, and bioinformatics also predict the relationship between the differentially expressed CHST3 and the protein CSPG4. Mounting studies report that CHST3 is a glycoprotein located in the extracellular matrix that catalyses chondroitin sulphide and also involved in cell differentiation and cell migration.^12^ CSPG4 is a glycoprotein that promotes cell proliferation and migration, whereas inhibiting nucleus pulposus axons from growing outward and inhibiting nucleus pulposus from collapsing.^13^, ^14^ However, the underlying molecular mechanisms of CHST3 and CSPG4 in CESCs of intervertebral disc degeneration remain unresolved and needs further investigation.

In the current study, we isolated CESCs from three patients with grade Ⅱ, Ⅲ and Ⅳ intervertebral disc degeneration, respectively. Then, the exact molecular mechanisms of CHST3 overexpression in CESCs to repair intervertebral disc degeneration by rat nucleus pulposus was explored one by one.

## 2 MATERIALS AND METHODS

### 2.1 Isolation of CESCs and expansion

Using clinically obtained degenerated intervertebral disc specimens (grades Ⅱ, Ⅲ and Ⅳ), the surrounding fibrous ring, nucleus pulposus and subchondral bone were cleaned under a dissecting microscope (×4 times). Rinse with phosphate buffer (PBS), remove the surrounding interstitial fluid and blood, obtain cartilage endplate tissue and digest the tissue with 0.2% type II collagenase + DMEM/F‐12 solution for 6 hours, and the cells were filtered by a 100 μM cell filter. After digestion of the cartilage endplate tissue, the filtrate was centrifuged at 1000 r/min for 10 minutes, the filtrate was discarded, and 10% FCS DMEM/F‐12 culture solution was added. Approximately, 2 × 10^6^ cells can be obtained from each specimen. The number of cells is counted on a blood cell counting plate. Placenta blue is used to assess the survival rate of the cells and observed under an inverted light microscope. In addition, the cells were cultured at 5% CO_2_ and 37°C. Cell exchange was performed once every 2‐3 days until the cells grew to the bottom of the culture flask, and then the cells were digested and expanded for culture.

### 2.2 Osteogenic induction with complete culture medium

Dexamethasone 3.925 mg, absolute ethanol 10 mL and final concentration 1 mM, aliquoted in 10 parts, 10 μL; vitamin C 17.612 mg, DMEM/F‐12 medium 10 mL and final concentration 10 mM aliquoted 10 parts, 500 μL; 3.0611 g of sodium phosphate, 9.1 mL of DMEM/F12 medium and final concentration of 1 M aliquoted into 10 portions, take 1 mL.

### 2.3 Chondroblastic induction with complete culture medium

Dexamethasone 0.1 μmol/L, bovine serum albumin (1.25 mg/mL), Vitamin C (37.5 μg/mL), sodium pyruvate (1 mmol/L), TGF‐B (10 ng/mL), β‐FGF (1 ng/mL) and ITS (6.25 pg/mL bovine insulin, 6.25 μg/mL transferrin).

### 2.4 GO and KEGG analysis of GSE15227 database

Based on the GSE15227 data analysis, the differentially expressed genes at different levels of disc herniation (Ⅱ, Ⅲ, Ⅳ) were obtained. *P* < .05, FDR > 1‐fold meaningful genes were selected for GO and KEGG analysis.

### 2.5 Immunohistochemistry (IHC)

The degenerated tissue of the freshly dissected intervertebral disc (<3 mm) was fixed with 2% paraformaldehyde and overnight at room temperature (RT) to prepare paraffin‐embedded sections for formalin fixation. These sections were stained after paraffin removal. The endogenous peroxidase activity was blocked by incubating the slices in 3% H_2_O_2_ methanol solution at room temperature for 10 minutes, and the endogenous peroxidase activity was blocked by incubating the slices at room temperature. The 100 L CHST3 or CSPG4, properly diluted, was applied to the slides and incubated at room temperature for 1 hour in a humidifying chamber. After washing with PBS FOR 3 TIMES, 100 L biotinylated antibodies were applied to slides and incubated at room temperature in a humidifying chamber for 30 minutes. 100 L SAV‐HRP Conjugates were applied to slides and incubated in a humidifying chamber at room temperature in dark for 30 minutes. The 100 LDAB substrate solution was applied to slides to reveal the colour of antibody staining. Allow colour development for less than 5 minutes until desired colour intensity was achieved. The tissue slides were dehydrated by four rounds of alcohol (95%, 95%, 100% and 100%) for five minutes each time. The colour of antibody staining in tissue sections was observed under the microscope.

### 2.6 Western blotting (WB)

The total proteins of the tissues or CESCs of intervertebral disc degeneration and the osteoblasts or chondroblasts from CESCs induction were extracted by strong RIPA lysis buffer. The concentration of total protein was determined by BCA assay. Then, the total protein was biologically treated at 100°C for 5 minutes to reduce and denature the samples. The 20 μg protein and molecular weight marker were added into the pores of the SDS‐PAGE gel. Transfer protein bands to the PVDF membranes and incubated with the primary antibodies of CHST3, CSPG4, OC, RUNX, aggrecan, collagen II, ELAVL1, VCAN, VASP, NCAN and OFD1 prepared by 1 × TBS and 5% non‐fat milk. After three times washing, the PVDF membranes were incubated at RT for 1 hour with the secondary antibodies combined with HRP. Finally, the ECL solution was applied to observe the protein bands and all results were statistically analysed by Image J.

### 2.7 Co‐immunoprecipitation (Co‐IP)

Prepare the tissue lysates of intervertebral disc degeneration in ice‐cold tissue lysis buffer and incubate on ice for at least 20 minutes. Sonicate the tissues in ice for at least five times. After high‐speed centrifugation, the supernatant was removed to a clean tube. The magnetic beads were resuspended several times by pipette and 50 μL beads were added to the supernatant and gently mix on ice. Add the primary antibodies of CSPG4 or CHST3 for IP assay to the above‐mentioned supernatant, which was equally divided into two parts. Incubate the mixture at 4°C for overnight under gentle agitation.

### 2.8 Immunofluorescence

Current identification of CESCs lacks specific surface markers. In 2006, the International Society for Stem Cell Therapy (ISCT) for cellular therapy proposes that stem cells should meet at least the following requirements: (1) Can adhere to growth in standard medium; (2) CD73, CD90 and CD105 must be overexpressed (>95%), and CD45, CD34, CD14 or CD11b, CD79a, CD19 and HLA II must be under expressed (below 2%) (3) and can differentiate into adipogenic, osteogenic and chondrogenic lines.^15^ The surface markers reported in CESCs are also mainly based on the CD phenotype including the following types: positive expression of CD90, CD73, CD105, STRO1, CD44 and CD166 and no expression of CD19, CD34, CD14, CD45 and HLA‐DR.^16^ In the current study, we detected the protein expression of CD90 and CD105 by immunofluorescence assay in intervertebral disc degeneration. In general, the deparaffinized sections were blocked with 0.1% BSA. They were then incubated overnight at 4°C with the primary fluorescein‐conjugated antibodies coupled with CD90 and CD105. After three times washing with pre‐cold PBS, the sections were observed under immunofluorescence microscopy. After elution for several times, the mixture was boiled at 100°C for 5 minutes. Finally, WB was performed to measure the effects of Co‐IP.

### 2.9 Flow cytometry for cell cycle

In this section, perform flow cytometry to identify the CESC’s characteristics. The isolated CESCs were prepared into single cell suspension. Then, add 5 μg/mL fluorescein‐conjugated primary antibodies of CD73, CD90 or CD105, respectively. Then, incubate at RT for at least 1 hour. After washing with ice‐cold PBS, analyse the single cell suspension on flow cytometer immediately.

### 2.10 Alizarin red staining

The CESCs were induced by osteogenic complete medium for 14 or 21 days, respectively. After washing with PBS and dehydrating in acetone, the cells were incubated with Alizarin red solution for 10 minutes and obtain the images under light microscopy. Usually, the cells will produce deep red–orange staining of calcium after 2 minutes.

### 2.11 TEM

The osteoblasts or chondroblasts induced from CESCs were fixed with 4% formaldehyde. After dehydration, the cells were embedded in bundles and roasted at 60°C for two days. Then, the cells were then ultrathin sectioned and collected on a grid, and stained with lead citrate for five minutes at RT. After rinsing with running tap water, the images were obtained under TEM.

### 2.12 Alcian blue staining

Cartilage tissue dewaxing slides induced by CESC for 14 or 21 days were stained in Alcian blue solution for 30 minutes. The slide was then rinsed in running tap water for 3 minutes. Dehydrate through 95% alcohol for three times and obtain the images under light microscopy.

### 2.13 CCK8 assay

The cell proliferation of osteoblasts or chondroblasts induced from CESCs were detected by CCK8 assay. The test compound was added into the cells incubated at RT for at least 2 hours. Then, the WST‐8 solution was added to the cells at RT in the darkness and incubated for at least four hours. Last but not the least, the absorbance was obtained at 460 nm and statistically analysed. This assay was repeated at least three times.

### 2.14 Colony formation assay for cell proliferation

The isolated CESCs were cocultured with bone marrow cells in the transwell plate, after transfection with ov‐CHST3 or sh‐CHST3; the cell proliferation was measured by colony formation assay. In general, the culture medium was removed and rinsed with PBS. Then, CESCs were incubated with 6.0% glutaraldehyde and 0.5% crystal violet at RT for at least 30 minutes. After rinsing with the running tap water, the colonies were calculated and statistically analysed.

### 2.15 Transwell assay for migration

Coculture of Transwell cells (non‐contact coculture of rat cartilage endplate stem cells and bone marrow cells in vitro Culture).

(1) Rat cartilage endplate stem cells were routinely digested with accutase and degenerative bone marrow cells with Trypsin. After 1.5 ~ 2.5 minutes, DMEM/F‐12 medium and 10% foetal bovine serum (FBS) was added to the suspension. Collect to centrifuge tube, 1000 rpm, 4 minutes collect cell, discard supernatant, cell with 10% standard foetal bovine. The serum was cultured in DMEM/F‐12 medium and the cells were counted. (2) Bone marrow cells suspensions were inoculated into transwell locules with about 1.5 × 10^5^ cells per well. Rat cartilage endplate stem cells were then injected into transwell cells in a suspension culture medium of about 2 mL. The number of cells in each hole is about 1.5 × 10^5^. Cell Culture medium is about 2 mL (note the two types of cells should be fully mixed before adding, and in the process of operation to avoid the generation of bubbles). (3) The above two kinds of cells were cultured overnight, and transwell's upper and lower chambers contained cell cultures. The upper chamber was then placed into lower chamber and cultured (note the upper chamber and the lower chamber). The contact surface cannot have the bubble), the total culture period every 3 days to change the liquid (half quantity change the liquid).

## 3 RESULTS

### 3.1 The differential gene expression was scientifically analysed by GO and KEGG in grade Ⅱ, Ⅲ and Ⅳ intervertebral disc degeneration

**FIGURE 1:**
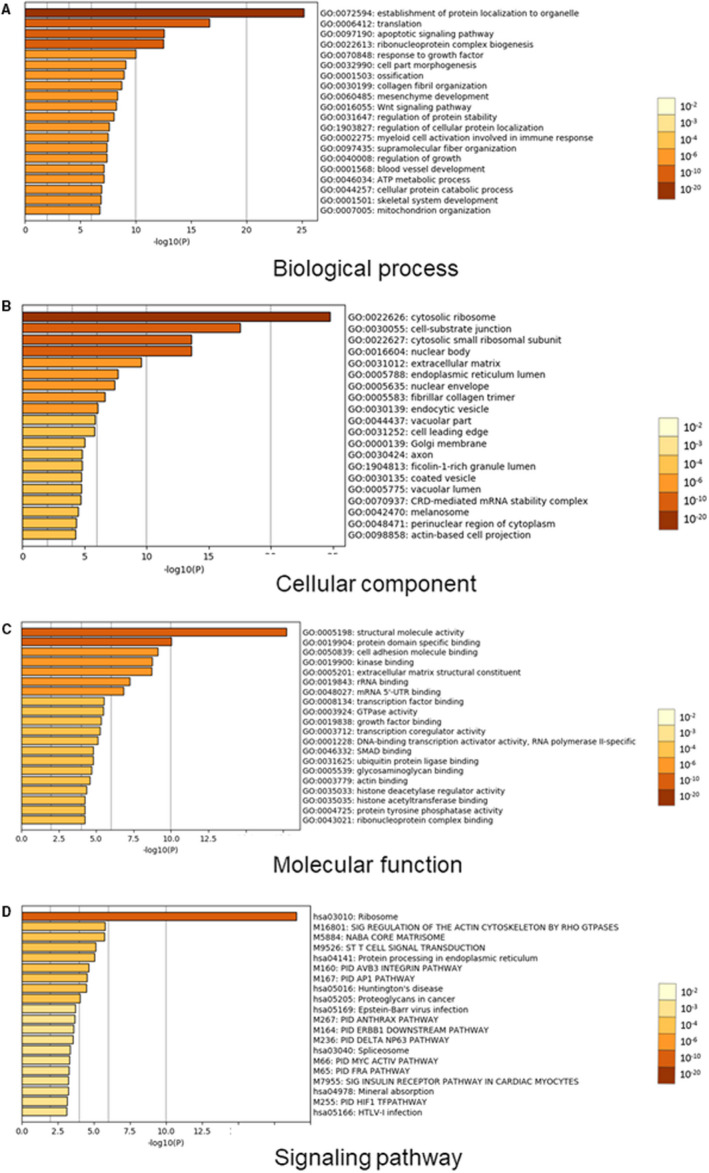
GO and KEGG analysis of the differential expression of grade Ⅱ, Ⅲ and Ⅳ intervertebral disc degeneration. Bioinformatic analysis demonstrated the biological process (A), cellular component (B), molecular function (C), signalling pathway (D) by GO and KEGG

Based on the bioinformatic database GSE15227, there was a great differential gene expression in intervertebral disc degeneration at different grades. This database was applied for GO and KEGG analysis, and *P* < .05 or FDR > 1 was set as the standards of meaningful genes. And we selected the top 2 differential genes CHST3 and CSPG4 as the aim of this study. These results demonstrated that there was a great difference in biological process (Figure 1A), cellular component (Figure 1B), molecular function (Figure 1C) and signalling pathway (Figure 1D) in grade Ⅱ, Ⅲ and Ⅳ intervertebral disc degeneration. This phenomenon showed a novel avenue for the following bioassays and provided some insights for the clinical strategies in the diagnosis or treatment for patients with intervertebral disc degeneration.

### 3.2 CHST3 and CSPG4 were differentially expressed and interacted in grade Ⅱ, Ⅲ and Ⅳ intervertebral disc degeneration

**FIGURE 2:**
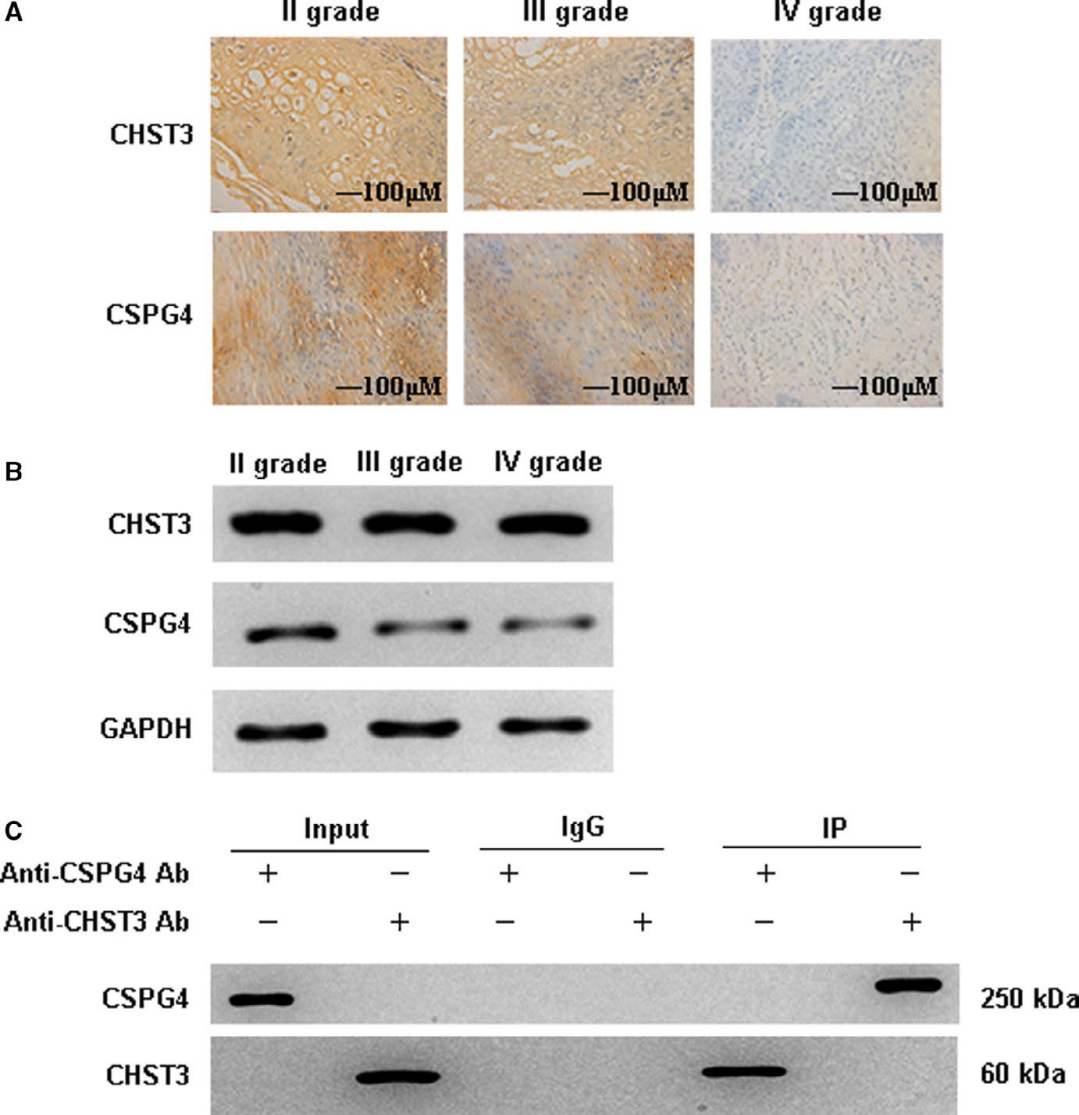
The protein expression of CHST3 and CSPG4 as well as their interaction in grade Ⅱ, Ⅲ and Ⅳ intervertebral disc degeneration. A‐B, IHC and WB analysis indicated the protein expression of CHST3 and CSPG4. Both proteins were lowly expressed in grade Ⅲ and Ⅳ tissues compared with those in grade Ⅱ. C, Co‐IP was performed to confirm the interaction between CHST3 and CSPG4

To further unveil the molecular mechanism of CHST3 and CSPG4 in intervertebral disc degeneration, IHC, WB and Co‐IP bioassays were applied to demonstrate their protein expression and interaction. The results of IHC showed that compared with grade II group, CHST3 and CSPG4 was almost remained unchanged in grade III group but both were significantly decreased in grade Ⅳ group (*P* < .05) (Figure 2A). WB assay further confirmed the results of IHC and proved that both proteins were lowly expressed in grade Ⅱ, Ⅲ and Ⅳ intervertebral disc degeneration (*P* < .05) (Figure 2B). Co‐IP analysis demonstrated that there was an interaction between CHST3 and CSPG4 in the tissues of intervertebral disc degeneration (Figure 2C). This phenomenon demonstrated that the differential expression of CHST3 and CSPG4 interacted with each other and their low expression contributed to the pathogenesis of grade Ⅱ, Ⅲ and Ⅳ intervertebral disc degeneration.

### 3.3 CD73, CD90 and CD105 were lowly expressed in grade Ⅱ, Ⅲ and Ⅳ intervertebral disc degeneration

**FIGURE 3:**
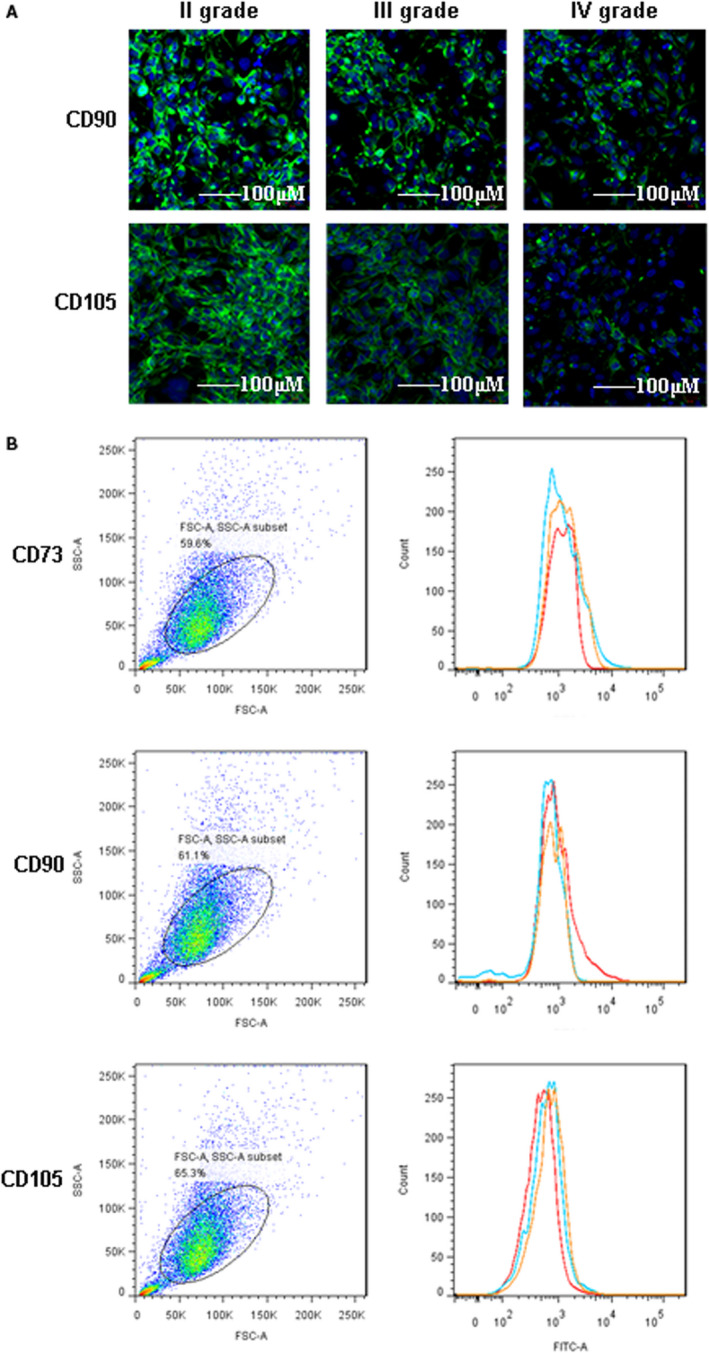
The expression features of CD73, CD90 and CD105 in grade Ⅱ, Ⅲ and Ⅳ intervertebral disc degeneration. A, Immunofluorescence assay indicated the level of CD90 and CD105 in grade Ⅱ, Ⅲ and Ⅳ intervertebral disc degeneration in CESCs. B, Flow cytometry demonstrated that the levels of CD73, CD90 and CD105 in CESCs were lower in grade Ⅲ and Ⅳ tissues than in grade Ⅱ tissues. CESCs were isolated from three clinical patients with intervertebral disc degeneration, respectively

Previous reports proved that CD73, CD90 and CD105 distinguished stem cells from different areas of intervertebral disc degeneration.^11^, ^16^, ^17^ In this study, immunofluorescence assay showed that CD90 and CD105 in grade Ⅲ and grade Ⅳ were significantly lower than those in grade Ⅱ in CESCs (*P* < .05) (Figure 3A). The isolated CESCs from three patients of grade Ⅱ, Ⅲ and Ⅳ groups with intervertebral disc degeneration were applied to detect the level of CD73, CD90 and CD105 by flow cytometry, and the results demonstrated that compared with grade Ⅱ group, the expression level of these proteins were obviously decreased to a large extent (*P* < .05) (Figure 3B). These results proved that CD73, CD90 and CD105 decreased with the aggregation of intervertebral disc degeneration, and these proteins could be used as the biomarkers for clinical diagnosis or treatment.

### 3.4 Osteogenic or chondroblastic induction changed the characteristics, proliferation, cell cycle and specific biomarkers of osteoblasts and chondroblasts after 14 or 21 days

**FIGURE 4:**
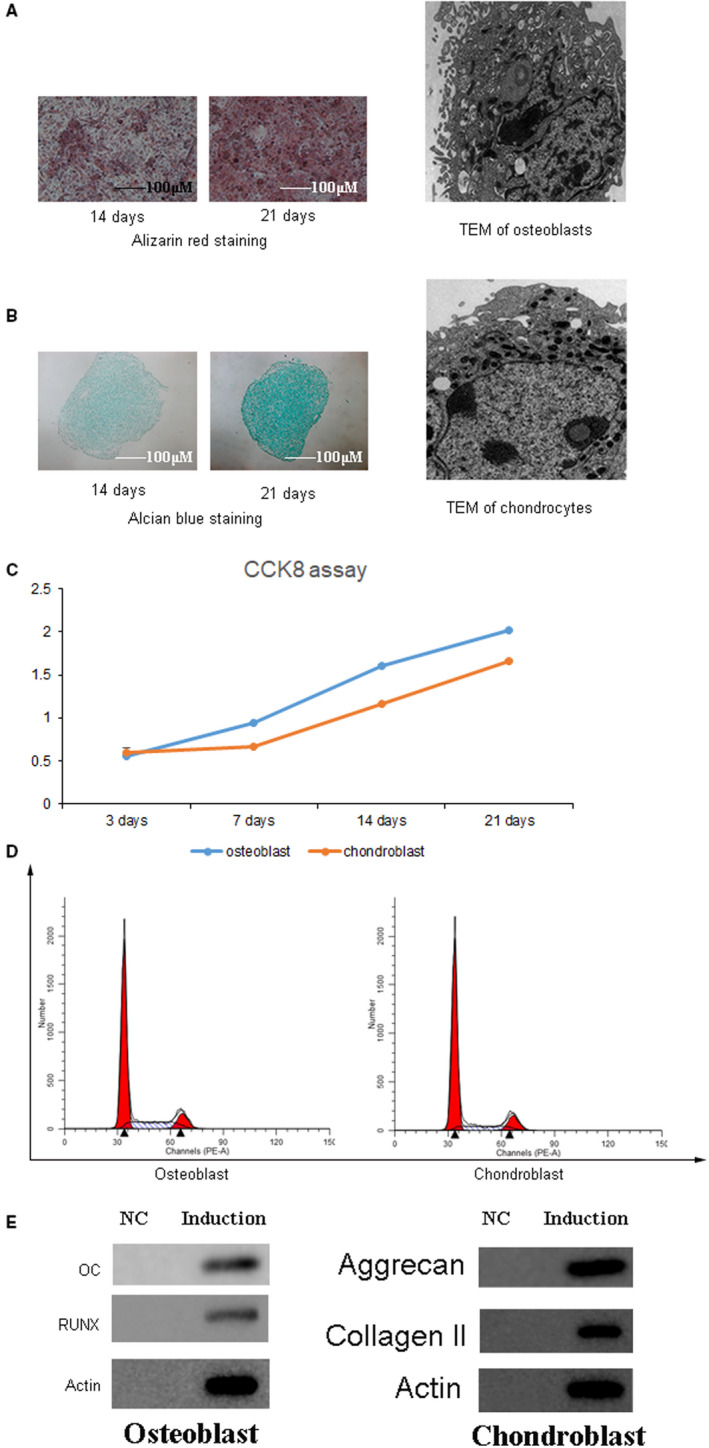
The characteristics, proliferation, cell cycle and specific biomarkers of osteoblasts and chondroblasts 14 or 21 d after induction. A, Alizarin red staining showed the successful induction of osteogenesis 14 or 21 d later. And TEM presented the intracellular features of osteoblast. B, Alcian blue staining indicated that the extracellular matrix of chondroblasts after chondroblastic induction for 14 or 21 d. TEM image showed the characteristics of chondroblasts. C, CCK8 assay measured the cell proliferation of osteoblasts and chondroblasts after induction for 3 d, 1 wk, 2 wk and 3 wk. The rate of cell proliferation increased in a time‐dependent way. D, Cell cycle of osteoblasts and chondroblasts was detected by flow cytometry after induction for 21 d. The cell content at G1 phase was significantly increased after induction when compared with other phases. E, WB showed the level of OC and RUNX in osteoblasts and the protein level of aggrecan, collagen Ⅱ in chondroblasts. The results showed that these specific biomarkers obviously increased after induction

It was well known that CESCs had a great potential for multiple differentiation,^18^ and in the current study, we mainly focused on the osteogenic and chondroblastic differentiation when CESCs were treated with induction medium for 14 or 21 days, respectively. The results of osteogenic induction showed that Alizarin red staining proved the successful induction of CESCs into osteoblasts; however, there was no difference between the 4‐day group and the 21‐day group. The image of TEM further presented the intracellular features of osteoblast induced from CESCs (Figure 4A). Meanwhile, the chondroblastic induction was confirmed by Alcian blue staining which had a deep staining on the extracellular matrix outside the chondroblasts. And there were significant differences between the 14‐day group and the 21‐day group. We also further confirmed the induction effects of CESCs into chondroblasts by TEM (Figure 4B). The cell proliferation of osteoblasts and chondroblasts after specific induction was demonstrated by CCK8 assay, and this result showed that the cell proliferation after induction was increased in a time‐dependent way, and there was significant difference between different time points such as 3 days, 1 week, 2 weeks and 3 weeks (*P* < .05) (Figure 4C). Furthermore, the cell cycle of osteoblasts and chondroblasts after induction from CESCs was measured by flow cytometry, and the results indicated that the cellular content at G1 phase (50.78% for osteoblasts and 58.36% for chondroblasts) was significantly higher than that at other phases including sub‐G1, S and G2/M phase (*P* < .05) (Figure 4D). WB assay confirmed the increased protein expression of OC and RUNX in osteoblasts and aggrecan and collagen Ⅱ in chondroblasts (Figure 4E). These results confirmed that after induction, CESCs were differentiated into osteoblasts or chondroblasts, which presented different characteristics, cell proliferation, cell cycle and the expression of specific biomarkers.

### 3.5 CHST3 affected the cell proliferation, protein profile, migration and cellular features of cocultured CESCs or bone marrow cells

**FIGURE 5:**
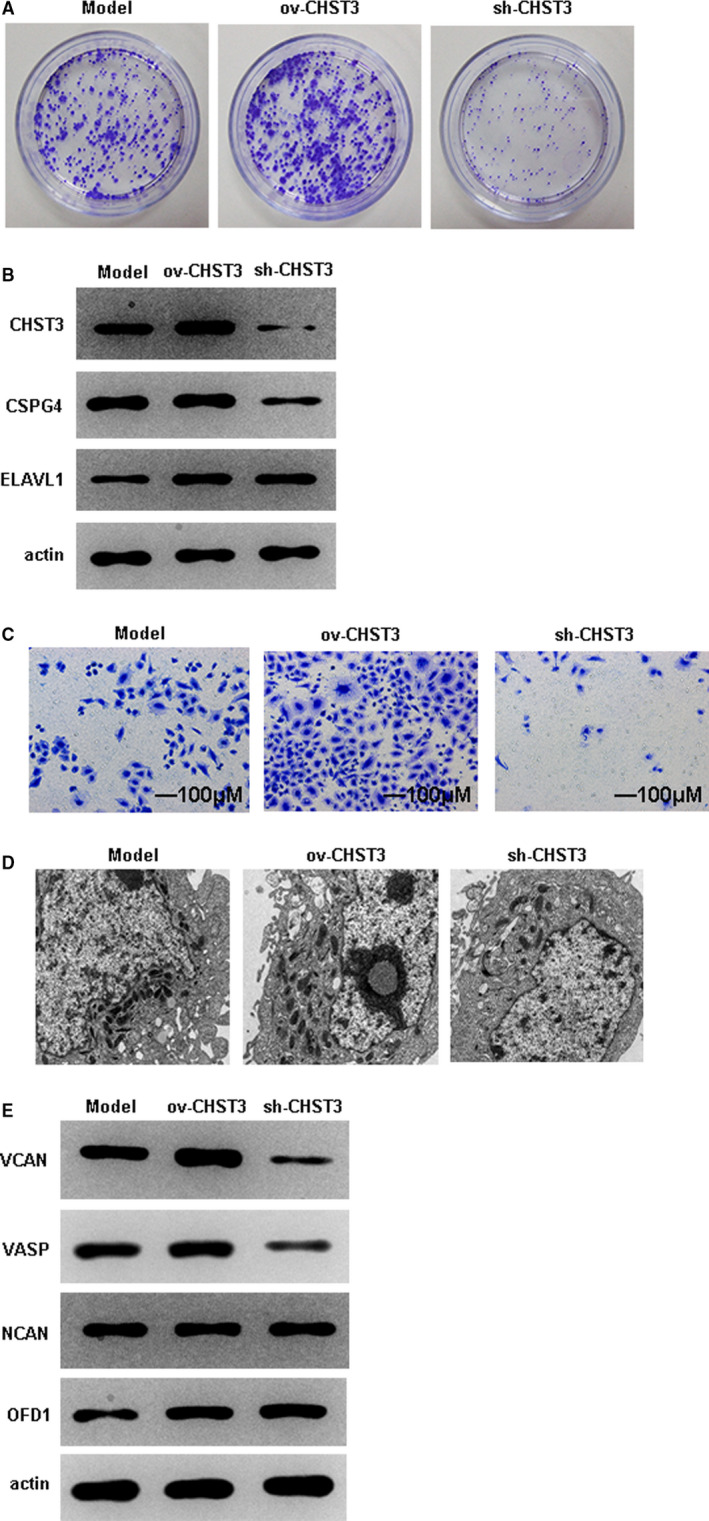
Influence of CHST3 on the cell proliferation, protein profile, migration and cellular features of CESCs or bone marrow cells when cocultured in transwell plate. A, Cell colony formation assay was applied to analyse the cell proliferation of CESCs. The isolated CESCs (upper layer) were cocultured with human bone marrow cells (lower layer) in the transwell plate, the overexpression plasmids or sh‐CHST3 plasmids were transfected into the CESCs for 72 h. This experiment was performed in triplicates and the results showed that the ov‐CHST3 group had a significant higher rate of cell proliferation than the sh‐CHST3 group. B, WB assay indicated the protein expression of CHST3, CSPG4 and ELAVL1 in CESCs after the treatment with ov‐CHST3 or sh‐CHST3. C, Transwell assay confirmed the migration of CESCs to bone marrow cells. The ov‐CHST3 group presented a significant higher migration ability than the sh‐CHST3 group. D, TEM image showed the intracellular characteristics of CESCs treated with ov‐CHST3 or sh‐CHST3, respectively. E, WB assay evaluated the protein level of VCAN, VASP, NCAN and OFD1 in bone marrow cells of the lower layer. CHST3 overexpression significantly elevated these proteins in bone marrow cells, whereas sh‐CHST3 completely reversed these effects

To further investigate the effects of CHST3 in CESCs, ov‐CHST3 and sh‐CHST3 plasmids were reconstructed by molecular technology according to the standard procedures. CESCs and bone marrow cells were cocultured in the transwell plates. CESCs were seeded in the upper layer, and the bone marrow cells were in the lower layer. When ov‐CHST3 were transfected into the CESCs, the cell proliferation was significantly increased; however, sh‐CHST3 reversed this effect oppositely (Figure 5A). What is more, the protein expression of CHST3, CSPG4 and ELAVL1 was obviously elevated in the group of ov‐CHST3 compared with the model group; however, the group of sh‐CHST3 presented an opposite phenomenon (Figure 5B). The migration of CESCs to bone marrow cells was significantly increased compared with that of model group, whereas sh‐CHST3 remarkably repressed the migration of CESCs in the transwell assay (Figure 5C). TEM further confirmed the cellular characteristics of bone marrow cells when they were treated with ov‐CHST3 or sh‐CHST3, respectively, and this result showed that there was no significant difference (Figure 5D). Last but not the least, the protein expression of VCAN, VASP, NCAN and OFD1 were significantly increased in bone marrow cells transfected with ov‐CHST3; however, the bone marrow cells showed a reverse effect when sh‐CHST3 was transfected (Figure 5E). These results indicated that CHST3 had a great influence on the cell proliferation, protein profile, migration and cellular features of cocultured CESCs or bone marrow cells.

## 4 DISCUSSION

Endogenous stem cells are the key to maintain homeostasis in tissues or organs.^19^ However, the role of CESCs in disc degeneration has not been well studied. Degenerative intervertebral disc is a kind of hypoxia, nutrient deficiency, slightly acidic, high osmotic pressure, high mechanical stress of the poor internal environment. Puluqiao et al found that the apoptosis of CESCs increased and the expression of apoptosis‐related proteins BNIP3, Bax and Bak upregulated under the condition of hypoxia and nutrient deficiency.^20^, ^21^ He et al found that low nutrient environment can induce high expression of BNIP3 protein in CESCs and induce apoptosis of CESCs through BNIP3 related signal pathway.^22^ Gene chip technology has been used to detect gene expression profiling changes in the process of CESCs cartilage differentiation and to identify potential targets for the treatment of CESCs degeneration; however, whether these gene changes are directly involved in the change in CESCs function needs to be verified by cell and animal experiments.^23^ Recent studies have shown that endogenous stem cells such as nucleus pulposus decrease in number and activity with ageing, suggesting that ageing of nucleus pulposus is very important in the degeneration of endogenous stem cells such as mesenchymal stem cell^24^, ^25^, ^26^; however, whether CESCs has similar phenomenon still needs further study. In this study, we performed bioinformatic analysis of GSE15227 database using grade Ⅱ, Ⅲ and Ⅳ intervertebral disc degeneration, and we choose CHST3 and CSPG4 as the main targets in the following investigation.

As the endogenous stem cells of intervertebral disc, CESCs has a natural advantage in adapting to the intervertebral disc environment, and it is easy to be obtained during operation. The recruitment and activation of endogenous stem cells to repair degenerated intervertebral discs avoid the iatrogenic injury of intervertebral discs that cannot be avoided by the transplantation of exogenous cells.^20^, ^27^ Wang et al transplanted nucleus pulposus, mesenchymal stem cell, a rabbit model of intervertebral disc degeneration was made by placing algae cells and bone marrow stem cells on alginate scaffolds; the results showed that CESCs had the best regeneration ability, and in vitro differentiation, and they also found that CESCs overmatched bone marrow stem cells, NPSCs and AFSCs in cartilage and osteogenesis; the results showed that CESCs as seed cells showed better application potential in intervertebral disc tissue engineering.^11^, ^28^ Transplanted CESCs compound hydroxyapatite subcutaneously into nude mice. Seven weeks after transplantation, CESCs was found to be osteogenic and differentiated. Sixteen weeks after implantation, CESCs was found to differentiate into chondroid‐like cells and promote disc repair.^29^ These studies suggest that CESCs can respond to a variety of biomaterials to further explore the downstream regulation mechanism can lay a foundation for the application of CESCs. We have compared the morphology, proliferation potential, cell cycle, immunophenotype, stem cell gene expressions and differentiation ability of CESCs, which demonstrates that CESCs may be a new cell source that may be more suitable for bone and cartilage repair. Agarose suspension culture is a chondrocyte selective culture system, in which chondrocytes are the only cell type to survive apart from tumour cells.

In the process of repairing intervertebral disc with tissue engineering technology, the most difficult problem is that the transplanted stem cells cannot adapt to the bad microenvironment of low oxygen, high osmotic and acid. CESCs has many advantages over exogenous stem cells, but its application is still facing difficulties. The main problems to be solved are as follows: (1) The specific phenotype of CESCs has not been determined, and the lack of efficient and specific sorting methods to separate CESCs from tissues has affected the further amplification and application of CESCs; (2) How to specifically induce CESCs to differentiate into hyaline cartilage but not into bone is an important problem in intervertebral disc regeneration; (3) There are some difficulties in the application of stem cells. Traditional stem cell applications include direct injection of stem cells, combined with scaffolds or gels as cell carriers implanted into tissues. The outer fibrous annulus of intervertebral disc is compact and the injection method will inevitably damage the outer fibrous annulus structure. In order to alleviate the injury to the fibre ring, the current use of thin needle injection. However, the chance of stem cell leakage is still inevitable. These leaky stem cells can develop heterotopic ossification, which in severe cases can compress nerve roots or crista. What is more, the survival rate of the injected stem cells was lower in the hostile environment of the degenerated disc, either in combination with the gel material or in the scaffold. In recent years, the concept of endogenous self‐repair has become a hot spot in the area of intervertebral disc. This study mainly performed to achieve tissue self‐repair by injecting chemokines or differentiation factors to recruit endogenous stem cells from the surrounding area stem cell nests. He et al found that CESCs can promote the proliferation of nucleus pulposus cells through paracrine pathway. Therefore, the study of endogenous biological factors to promote the CESCS activity can not only promote the repair of cartilage endplates, but also through its secretion of cytokines can repair the nucleus pulposus tissue.

The cytokines secreted by BMSCs can act on bone marrow cells, and bone marrow cells also create a microenvironment for BMSCs. The system simulates the microenvironment of bone marrow cells in vivo, which is closer to the internal environment of human body than the traditional three‐dimensional culture method and is more convenient for the observation and study of cell morphology. But, this is only in vitro culture. Next, we will transplant the co cultured BMSCs into the degenerative intervertebral disc and observe their changes, so as to lay a solid foundation for further treatment of intervertebral disc degeneration.

The discovery of CESCs is of great importance for the repair of disc degeneration in the future. CESCs not only has a good ability of proliferation and differentiation, but also has a higher ability of proliferation and differentiation, and these stem cells show better adaptability in the degenerative disc environment. Therefore, it has a good application prospect in the research of intervertebral disc regeneration. It also promotes the proliferation and migration of CESCs in the degenerated cartilage endplate to the injured site; the completion of tissue self‐repair is important for the future research of intervertebral disc repair and regeneration Direction. We believe that with the development of research on CESCs in disc degeneration and repair in‐depth, the biological treatment of intervertebral disc may usher in a new breakthrough.

## CONFLICT OF INTEREST

The authors declare that they have no competing interests.

## AUTHOR CONTRIBUTION

**Yunzhi Guan:** Conceptualization (equal); Data curation (equal); Formal analysis (equal); Investigation (equal); Methodology (equal); Resources (equal); Software (equal); Supervision (equal); Validation (equal); Visualization (equal); Writing‐original draft (equal); Writing‐review & editing (equal). **Chi Sun:** Conceptualization (equal); Data curation (equal); Formal analysis (equal); Investigation (equal); Methodology (equal); Resources (equal); Software (equal); Supervision (equal); Validation (equal); Visualization (equal); Writing‐original draft (equal); Writing‐review & editing (equal). **Fei Zou:** Conceptualization (equal); Data curation (equal); Formal analysis (equal); Funding acquisition (equal); Investigation (equal); Methodology (equal); Resources (equal); Software (equal); Supervision (equal); Validation (equal); Visualization (equal); Writing‐original draft (equal); Writing‐review & editing (equal). **Hongli Wang:** Formal analysis (equal); Investigation (equal); Project administration (equal); Resources (equal); Software (equal); Supervision (equal); Validation (equal); Visualization (equal). **Feizhou Lu:** Data curation (equal); Formal analysis (equal); Investigation (equal); Methodology (equal); Software (equal); Supervision (equal); Validation (equal); Visualization (equal). **Jian Song:** Data curation (equal); Formal analysis (equal); Investigation (equal); Resources (equal); Software (equal); Validation (equal). **Siyang Liu:** Formal analysis (equal); Investigation (equal); Methodology (equal); Resources (equal); Software (equal); Supervision (equal). **Xinlei Xia:** Data curation (equal); Formal analysis (equal); Methodology (equal); Resources (equal); Software (equal); Supervision (equal). **Jianyuan Jiang:** Formal analysis (equal); Funding acquisition (equal); Resources (equal); Software (equal); Supervision (equal); Visualization (equal). **Xiaosheng Ma:** Formal analysis (equal); Investigation (equal); Software (equal); Supervision (equal); Validation (equal).

## ETHICS APPROVAL AND CONSENT TO PARTICIPATE

The research protocol was reviewed and approved by the Ethical Committee and Institutional Review Board of the Huashan Hospital, Fudan University.

## CONSENT TO PUBLISH

All of the authors have consented to publish this research.

## Data Availability

The data are free access to available upon request.
